# Frequency and clinical features of treatment-refractory myasthenia gravis

**DOI:** 10.1007/s00415-019-09667-5

**Published:** 2019-12-11

**Authors:** Jakob Rath, Ines Brunner, Matthias Tomschik, Gudrun Zulehner, Eva Hilger, Martin Krenn, Anna Paul, Hakan Cetin, Fritz Zimprich

**Affiliations:** Department of Neurology, Medical University of Vienna, Währinger Gürtel 18-20, 1090 Vienna, Austria

**Keywords:** Myasthenia gravis, Outcome, Refractory disease, Treatment

## Abstract

**Background:**

To investigate the frequency and characterize the clinical features of treatment-refractory myasthenia gravis in an Austrian cohort.

**Methods:**

Patient charts of 126 patients with generalized myasthenia gravis and onset between 2000 and 2016 were analyzed retrospectively. Patients were classified as treatment-refractory according to strict, predefined criteria. These mandated patients being at least moderately symptomatic (i.e., MGFA class III) or needing either maintenance immunoglobulins or plasma exchange therapy for at least 1 year in spite of two adequately dosed immunosuppressive drugs. Clinical features and outcome at last follow-up were compared to treatment-responsive patients.

**Results:**

14 out of 126 patients (11.1%) met these criteria of treatment-refractory myasthenia gravis. Treatment-refractory patients had more frequent clinical exacerbations and more often received rescue treatments or a further escalation of immunosuppressive therapies. They also remained more severely affected at last follow-up. An early onset of myasthenia gravis was associated with a higher risk for a refractory course.

**Conclusion:**

A small subgroup of patients with generalized myasthenia gravis do not respond sufficiently to standard therapies. Refractory disease has considerable implications for both patients and health care providers and highlights an unmet need for new treatment options.

## Introduction

Myasthenia gravis is an autoimmune disease of the neuromuscular junction with a prevalence of around 16 per 100,000 [1]. Patients are grouped according to the age at onset, presence of a specific antibody, thymus pathology, and distribution of symptoms [2]. The majority of patients (approximately 80%) have antibodies against the nicotinic acetylcholine receptor (AChR), while in a small subset of patients antibodies against muscle-specific receptor tyrosine kinase (MuSK), lipoprotein-related protein 4 (LRP4) or other postsynaptic structures of the neuromuscular junction are detected. In about 5% of patients, no antibodies are found. Additionally, paraneoplastic disease can occur in patients with thymoma that leads to generalized thymoma-associated myasthenia gravis with the detection of AChR-antibodies in nearly all patients [3, 4].

The natural, untreated course of myasthenia gravis has been associated with a high mortality and a persistence of symptoms in most patients [5, 6], but the introduction of immunosuppressive treatments, thymectomy in selected patients, modern intensive care medicine as well as the availability of rescue treatments such as intravenous immunoglobulins (IVIG), plasma exchange therapy (PLEX) or immunoadsorption (IA) has greatly improved the outcome across all subgroups of patients [7]. However, approximately 10–15% of patients still show a poor response to available standard treatments and consequently continue to suffer from disabling symptoms. They also experience frequent disease exacerbations leading to a reduced quality of life and frequent admissions to hospitals and emergency departments [8–11]. In addition, the necessary treatment with high-dose immunosuppressive drugs is often associated with side effects, which negatively affects patients’ quality of life.

So far, only a few studies have specifically addressed the characteristics of treatment-resistance myasthenia gravis patients [12–14]. It is also an open question which factors predispose patients to a refractory disease course with some observations suggesting an early onset, female gender, an association with thymoma or the presence of MuSK-antibodies as risk factors [13, 14]. Given the unmet clinical needs in treatment-refractory patients, a further characterization and definition of this subgroup is clearly warranted to recognize and select patients early for a targeted management with modern immunosuppressive drugs [15, 16].

The aim of this retrospective study was to evaluate the frequency of treatment-refractory disease courses among patients with generalized myasthenia gravis according to a strict definition and assess the clinical features of these patients.

## Methods

### Patients

We retrospectively investigated charts of patients with onset of myasthenia gravis between 2000 and 2016, who were treated at our tertiary neuromuscular center at the Department of Neurology of the Medical University of Vienna. We included only patients with sufficient follow-up data of at least 2 years and generalized myasthenia gravis within the first year after onset. Diagnostic criteria for myasthenia gravis consisted of typical myasthenic symptoms in combination with myasthenia gravis-related antibodies, or in seronegative patients either pathological repetitive nerve stimulation with a decrement over 10%, a positive edrophonium chloride test, or documented clinical improvement following pyridostigmine treatment. Disease severity was retrospectively assessed at documented time points using the criteria by the Myasthenia Gravis Foundation of America (MGFA) class [17]. Ethical approval was obtained from the Ethics Committee of the Medical University of Vienna.

### Outcome measures

The primary outcome measure was the occurrence of treatment-refractory MG.

We defined treatment-refractory myasthenia gravis at the earliest 2 years after diagnosis as soon as the following conditions were met:

1. Persistent moderate to severe myasthenic symptoms (i.e., ≥ MGFA class III) for the last 12 months *OR*
2. MGFA class < III but requirement of regular maintenance treatment with IVIG or PLEX/IA for the last 12 months *in combination with*
  Treatment with at least 2 concurrent long-term immunosuppressive drugs at adequate doses for the last 12 months.

Long-term immunosuppressive drugs included all conventional therapies including corticosteroids, azathioprine, mycophenolate-mofetil, and tacrolimus. The average prednisone-equivalent dose had to be at least ≥ 5 mg daily. Escalation treatment with rituximab and pulsed cyclophosphamide given according to standard regimens was regarded as equivalent to the treatment with 1 conventional immunosuppressive drug for 12 months.

Secondary outcome measures were MGFA class, MGFA postintervention status for asymptomatic patients, treatment at last follow-up and all-cause mortality. Additionally, the number of myasthenic crises (MGFA class V) and severe exacerbations of myasthenic symptoms (defined as clinical deterioration requiring acute medical intervention or inpatient treatment but without the need for mechanical ventilation) was assessed. Furthermore, the number of rescue treatments with IVIG or PLEX/IA during the course of disease and occurrence of severe side effects of immunosuppressive treatments was analyzed.

### Statistical analysis

Statistical analysis was performed with SPSS 24 software package (IBM, Corp. Released 2016. IBM SPSS Statistics for Macintosh, Version 24.0. Armonk, NY: IBM Corp.). Baseline variables as well as outcome measures of treatment-refractory and treatment-responsive patients were compared using the Student’s *t* test or Mann–Whitney *U* test for continuous variables and Chi-squared test for categorical variables.

Multivariate logistic regression analyses were used to test for clinical variables associated with the occurrence of treatment-refractory myasthenia gravis. Covariates were selected according to clinical meaningful aspects. The following covariates and their interactions were included in the final model: EOMG vs. LOMG, sex, antibody status, and thymus histology indicating thymoma-associated myasthenia gravis. A *p* value of ≤ 0.05 was considered statistically significant; correction for multiple comparisons for analyses of secondary outcome measures was done using Bonferroni correction resulting in a *p* value of ≤ 0.004.

## Results

**Fig. 1:**
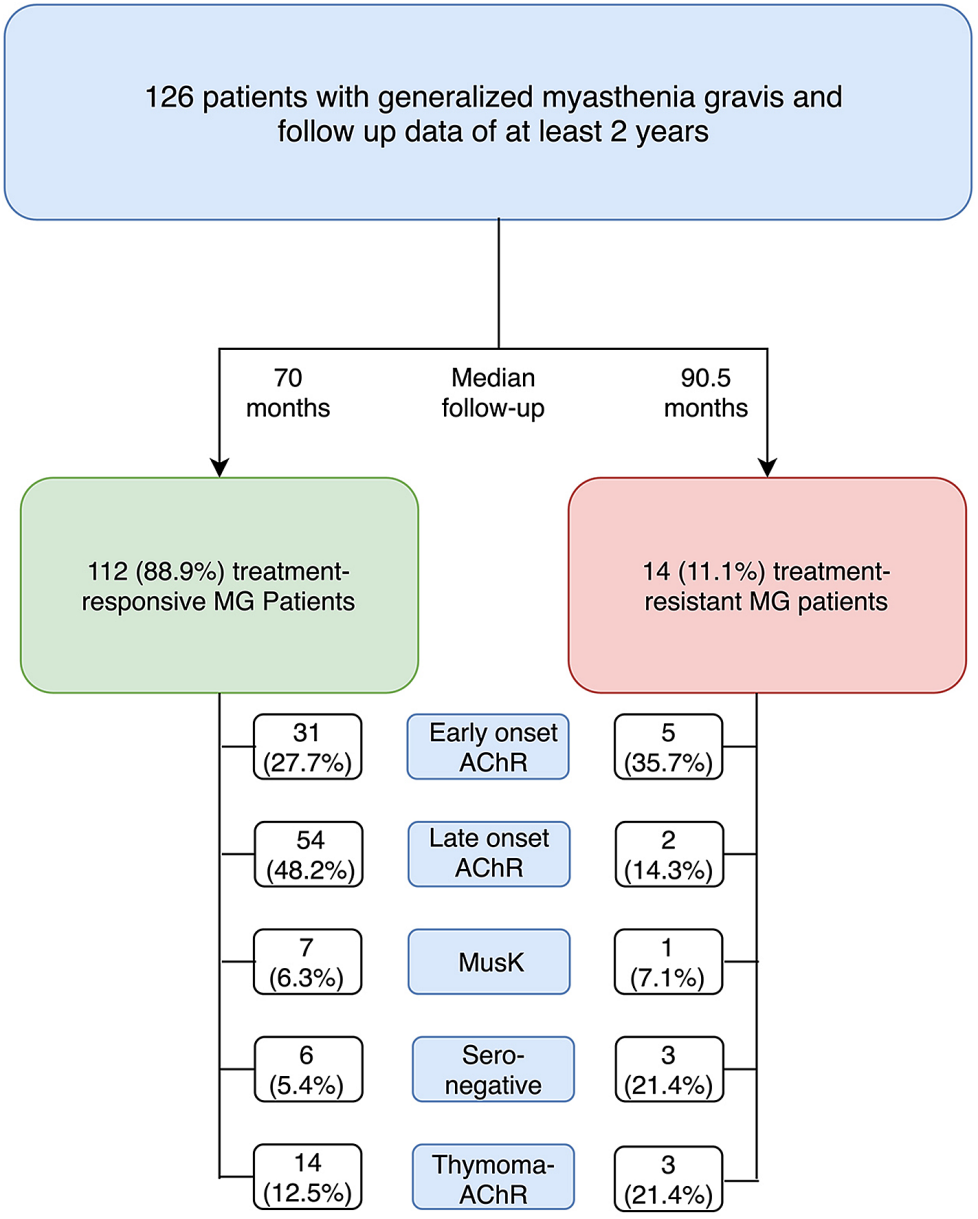
Rates of treatment-refractory MG and treatment-responsive MG according to subgroups suggested by Gilhus et al. [2]; *AChR* denotes acetylcholine receptor, *MG* Myasthenia gravis and MuSK muscle-specific tyrosine kinase

126 patients (54 men, 72 women; median age at onset 49.5, interquartile range (IQR) 37, total range (13–85) were analyzed retrospectively. Of these, 14 (11.1%) patients were classified as treatment-resistant myasthenia gravis (see Fig. 1 for distribution of patients according to the subgroups proposed by Gilhus et al. [2] and Table 3 for detail description of individual patient characteristics).

9 of the 14 patients met the criteria because of persistent myasthenic symptoms and 5 patients because they required maintenance IVIG or PLEX/IA treatment. Patients met the criteria of treatment-resistant myasthenia gravis after a median of 44.5 months (IQR 40 months, total range 24–197 months). Of the 14 treatment-refractory patients, 8 were diagnosed in the first half (01 January, 2000–31 June, 2008) and 6 in the second half (01 July, 2008–31 December, 2016) of the analyzed time period (*p* = 0.12).

Baseline variables for all patients as well for the treatment response-groups are shown in Table 1. Baseline variables did not differ significantly between the two groups regarding time from onset to initiation of immunosuppressive treatment, AChR-antibody titer, MGFA class at onset, major comorbidities, time to immunosuppressive treatment initiation, thymectomy status or thymus histology. However, patients in the treatment-refractory group were younger (median age 33 vs 50.5, *p* = 0.035) and were more often classified as EOMG than LOMG. Multivariate regression analysis confirmed the statistically significant association of EOMG with a higher chance of developing treatment-refractory MG (*p* = 0.011, OR 8.35, 95% CI 1.64–42.64). Additionally, male sex was also associated with treatment failure in the multivariate analysis (*p* = 0.011, OR 6.05, 95% CI 1.51–24.15), but not in the univariate analysis (*p* = 0.26). Antibody status and presence of thymoma in thymus histology were not significantly associated with treatment-refractory myasthenia gravis in the multivariate analyses.

**Table 1:** Baseline characteristics

	All patients (*n* = 126)	Treatment-refractory (*n* = 14)	Treatment-responsive (*n* = 112)	*p* value*
Sex				0.25
Male	54 (42.9%)	8 (57.1)	46 (41.1%)	
Female	72 (57.1%)	6 (42.9%)	66 (58.9%)	
Median age	49.5 years (IQR 37)	33 years (IQR 25)	50.5 (IQR 38)	**0.035**^‡^
EOMG (< 50 years)	63 (50%)	11 (78.6%)	52 (46.4%)	**0.023**^‡^
Antibodies				0.085
AChR	109 (86.5%)	10 (71.4%)	99 (88.4%)	
MuSK	8 (6.3%)	1 (7.1%)	7 (6.3%)	
Seronegative**	9 (7.1%)	3 (21.4%)	6 (5.4%)	
Median AChR-Ab titer at onset	17.2 (IQR 27.85)	9.9 (IQR 16.6)	17.5 (IQR 29.2)	0.42
MGFA class at onset				0.96
1	31 (24.6%)	4 (28.6%)	27 (24.1%)	
2	77 (61.1%)	9 (64.3%)	68 (60.7%)	
3	13 (10.3%)	1 (7.1%)	12 (10.7%)	
4	3 (2.4%)	0	3 (2.7%)	
5	2 (1.6%)	0	2 (1.8%)	
Max. MGFA class				** < 0.000**^**‡**^
2	68 (54%)	0	68 (60.7%)	
3	31 (24,6%)	9 (64.3%)	22 (19.6%)	
4	18 (14.3%)	4 (28.6%)	14 (12.5.%)	
5	9 (7.1%)	1 (7.1%)	8 (7.1%)	
Thymectomy	68 (54%)	10 (71.4%)	58 (51.8%)	0.16
Thymus histology				0.62
Normal	29 (42.7%)	3 (30%)	26 (44.8%)	
Hyperplasia	20 (29.4%)	4 (40%)	16 (27.6%)	
Thymoma	17 (25%)	3 (30%)	14 (24.1%)	
No data	2 (2.9%)	0	2 (3.5%)	
Severe comorbidity	23 (18.3%)	3 (21.4%)	20 (17.9%)	0.74
Median time onset to IST (months)	3 (IQR 9)	2.5 (IQR 9)	3 (IQR 9)	0.61

Baseline characteristics of all patients and comparison of treatment-refractory and treatment-responsive patients

*AChR* denotes acetylcholine receptor, *MG* myasthenia gravis, *MGFA* Myasthenia Gravis Foundation of America, *MuSK* muscle-specific tyrosine kinase, *EOMG* early-onset myasthenia gravis, *IS* immunosuppressive, *IQR* interquartile range, *NA* not applicable

^*^p values were obtained with the Mann–Whitney *U* or Student’s *t* test (for continuous variables) and the Chi-square test (for categorical variables) as appropriate

^**^Of the 10 seronegative patients 4 [2 of whom were treatment-refractory) were tested negative for antibodies against AChR by radioimmunoassay (RIA)], MuSK, LRP4 and AChR by cell binding assay, 5 (one of whom were treatment-refractory) against AChR (RIA) and MuSK and 1 against AChR (RIA) only

^‡^Statistically significant

Results of secondary outcome measures are shown in Table 2. Treatment-refractory patients had significantly higher maximum MGFA classes during their course of disease, 85.7% of these patients received escalation treatment with either rituximab or cyclophosphamide at some point and they required more rescue treatments with IVIG or PLEX/IA (median 3 times vs. 0.5, *p* = 0.002). At the last follow-up after a median of 70 months for the treatment-responsive group and 90.5 months for treatment-resistant group, patients who had met the criteria for treatment-refractory myasthenia gravis at any point during the course of disease still had higher MGFA classes than treatment-responsive patients. Furthermore, all treatment-refractory patients required ongoing immunosuppressive treatment and 35.7% continued to receive additional maintenance treatment with IVIG or PLEX/IA. While we found no statistically significant differences in occurrence of myasthenic crisis or mortality, treatment-refractory patients had significantly higher numbers of severe myasthenic exacerbations. Treatment-related side effects occurred in 37.5% of treatment-responsive patients compared to 57.1% in treatment-refractory patients, but the difference was not statistically significant.

**Table 2:** Results of secondary outcome measures

	Treatment-refractory MG (*n* = 14)	Treatment-responsive MG (*N* = 112)	*p* value*
Myasthenic crisis	2 (14.3%)	8 (7.1%)	0.351
Severe exacerbation	9 (64.3%)	30 (26.8%)	**0.004**^‡^
Mortality	2 (14.3%)	10 (8.9%)	0.52
MGFA at last FU			** < 0.000**^‡^
Asymptomatic	1 (7.1%)	77 (68.8%)	
1	3 (21.4%)	7 (6.3%)	
2	6 (42.9%)	24 (21.4%)	
3	4 (28.6%)	4 (3.6%)	
4	0	0	
5	0	0	
MGFA-PIS at last FU**			NA
CSR		12 (10.7%)	
PR		19 (17%)	
MM-0		0	
MM-1		2 (1.8%)	
MM-2		6 (5.4%)	
MM-3	1 (7.1%)	38 (33.9%)	
Median time onset to FU	90.5 months (IQR 104)	70 months (IQR 67)	0.047
Median number of rescue treatments per patient with IVIG, PLEX or IA	3 (IQR 6)	0.5 (IQR 1)	**0.002**^‡^
Median number of different IS treatments	3 (IQR 1)	2 (IQR 1)	** < 0.000**^‡^
Escalation IS treatment***	12 (85.7%)	6 (5.4%)	** < 0.000**^‡^
Side effects of IS treatment	8 (57.1%)	42 (37.5%)	0.23
Treatment at last FU			
Pyridostigmine	12 (85.7%)	73 (65.2%)	0.12
IS treatment	14 (100%)	84 (75%)	0.034
Maintenance IVIG/PLEX/IA	5 (35.7%)	3 (2.7%)	** < 0.000**^‡^

Results of secondary outcome measures. Significance level after correction for multiple comparisons (Bonferroni correction) is *P* ≤ 0.004

*CSR* denotes complete stable remission, *FU* follow-up, *IA* immunoadsorption, *IS* immunosuppressive, *IVIG* intravenous immunoglobulins, *MG* myasthenia gravis, *MGFA* Myasthenia Gravis Foundation of America, *MM* minimal manifestation, *NA* not applicable, *PIS* postintervention status, *PLEX* plasma exchange therapy and PR pharmacologic remission

*p values were obtained with the Mann–Whitney *U* or Student’s *t* test (for continuous variables) and the Chi-squared test (for categorical variables) as appropriate

**16 patients in the treatment-responsive group did not meet the time criterion (duration of at least 1 year) of MGFA-PIS definitions

***Escalation IS treatment was defined as treatment with rituximab or cyclophosphamide

^‡^Statistically significant

## Discussion

In this study, we retrospectively investigated the frequency and clinical features of patients with treatment-refractory generalized myasthenia gravis. We found that 11.1% of our study population met the respective criteria indicating persistent myasthenic symptoms despite adequate treatment with at least two concurrent immunosuppressive drugs, usually steroids plus a steroid-sparing agent for at least the last year before assessing resistance. The other main finding of the study was that patients who became treatment-refractory during their course of disease, still had a worse outcome at last follow-up despite more aggressive ensuing treatment.

In comparison with previous studies, the percentage of treatment-refractory myasthenia gravis in our cohort is within the reported range of 9.2–14.8% [12–14]. In contrast to these studies, however, we excluded patients with pure ocular myasthenia gravis, thus our findings are applicable only to patients with generalized symptoms developing within the first year of the disease. As detailed above, we used a strict definition of treatment-refractory myasthenia gravis based on the duration of treatment and symptoms, which partly differs from previously suggested criteria [9, 12–15]. Most importantly, we limited our definition to moderate to severe patients (i.e., a MGFA class of III or more). While we acknowledge that patients with persistent milder symptoms might also be affected to a relevant degree in their abilities of daily living and that these patients are potentially better captured by more lenient proposed definitions of refractory myasthenia gravis [18], we believe that our criteria complement previous criteria because they specifically target refractory patients who are clinically severely affected despite aggressive immunosuppressive treatment.

It is noteworthy that we also excluded patients who did not tolerate an adequate immunosuppressive therapy in contrast to definitions suggested by other authors [9]. We believe that these patients represent a different subgroup and should therefore be investigated separately as treatment-intolerant rather than refractory.

Our results suggest that patients with an onset of the disease before the age of 50 years might have a higher chance of becoming treatment-refractory than older patients. This confirms the results of Suh et al. [13] and is indirectly supported by a previous study reporting better outcome in older patients [7] but in contrast to other studies [19]. Varying rates of immunosuppressive treatments in older patients at different neuromuscular centers might explain the conflicting results. In comparison to previous reports that investigated treatment-refractory patients [13, 14], our findings differ insofar as we could not find an association of thymoma-associated myasthenia gravis, presence of MuSK-antibody or female sex with treatment-refractory myasthenia gravis. However, given the small number of patients in the respective subgroups, these findings should be interpreted with caution. The question if male or female sex is a risk factor, particularly warrants further investigation given the discrepancy between the multivariate analysis in our study suggesting an association of male sex with treatment-refractory myasthenia gravis, which was not evident in the univariate analysis, and previous outcome studies reporting no influence of sex [7, 19]. Nonetheless, our finding that MuSK-antibody myasthenia gravis is not associated with a higher risk for refractory myasthenia gravis most likely reflects recent changes in treatment practices, which favor early treatment with rituximab leading to improved outcomes in this subgroup [20].

The frequency of myasthenic crises in patients without refractory disease was similar to previously reported cohorts [11] with 7.1% of patients experiencing at least one episode. Likewise, the rate of myasthenic crises in the refractory myasthenia gravis group (14.3%) was only slightly lower than the reported number for treatment-refractory patients in the REGAIN study (18%, cohort limited to AChR antibody positive myasthenia gravis) and the study by Engel-Nitz et al. (21.3%) [11, 15]. Furthermore, the high number of severe exacerbations (64.3%) in our cohort of treatment-refractory patients was comparable to the REGAIN population, where 78% of patients reported any exacerbation of myasthenia gravis before enrollment and to the 71.2% reported by Engel-Nitz and colleagues.

Concerning treatment, refractory patients expectedly received on average more immunosuppressive drugs and also more escalation therapies, but the time to immunosuppressive treatment initiation did not differ between the groups. Since previous studies showed an association of a shorter time to diagnosis with better remission, additional studies are needed to investigate if faster and more aggressive treatment approaches are beneficial in certain myasthenia gravis subgroups. The frequent exacerbations in treatment-refractory patients were also accompanied by a substantially higher number of rescue treatments. However, despite the higher number of both standard and escalation immunosuppressive treatments, only one patient in the treatment-refractory group was clinically asymptomatic at last follow-up, all patients required ongoing immunosuppressive treatments and about a third continued to be dependent on additional maintenance treatment with either IVIG or PLEX/IA, further emphasizing the enormous impact on health care systems and the individual patient.

This study has some inherent limitations. First, due to the retrospective design, patient data regarding clinical deterioration could have been missed, but to minimize this effect we only included patients with sufficient clinical information available. Second, our cohort represents a myasthenia gravis population at a specialized tertiary department, therefore we cannot exclude a selection bias towards more severely affected patients. Furthermore, time to last follow-up was slightly shorter in the treatment-responsive group, thus it cannot be excluded that some patients of this group would have become treatment-refractory after the last follow-up. However, most patients met the criteria considerably earlier than at the last follow-up, arguing against a relevant bias (Table 3). Finally, the definition of treatment-refractory myasthenia gravis was clinically based on retrospectively rated MGFA classes, which partly depends on subjective assessment of clinical symptoms. Therefore, future studies are necessary to define treatment-refractory myasthenia gravis based on quantitative scores (i.e., myasthenia gravis composite score or the quantitative myasthenia gravis score [17]).

**Table 3:** Characteristics of treatment-refractory patients

	Sex	Age at onset	Antibody	Thymectomy	Histology	Time to refractory MG (mos.)	IS treatment at refractory timepoint	MGFA class at last FU
1	m	73	AChR	No	NA	60	CS 5 mg, MMF 1000 mg, CP (1g4w)	IIIa
2	m	59	AChR	No	NA	56	CS 75 mg, CP (1 g q4w), IA/PLEX	IIIb
3	m	41	MuSK	No	NA	24	CS 18.8 mg, TC 6 mg, IA/PLEX	IIb
4	m	54	AChR	Yes	Thymoma	24	CS 12.5 mg, AZA 150 mg, IA/PLEX	0 (MM3)
5	f	22	SN	Yes	Hyperplasia	197	CS 10 mg, RTX (375 mg/m^2^ BSA)	IIa
6	f	35	AChR	Yes	Thymoma	74	CS 25 mg, MMF 2000 mg	IIa
7	f	27	AChR	Yes	Normal	24	CS 8.75 mg, AZA 150 mg, IA/PLEX	IIb
8	m	13	AChR	Yes	Hyperplasia	158	MMF 150, RTX (375 mg/m^2^ BSA), IA/PLEX	I
9	m	49	AChR	No	NA	52	CS 25 mg, AZA 200 mg, IVIG	I
10	m	28	SN	Yes	Hyperplasia	49	CS 25 mg, MMF 2000 mg, IVIG	I
11	f	27	AChR	Yes	Normal	24	CS 25 mg, AZA 100 mg, IVIG	IIb
12	m	48	AChR	Yes	Thymoma	25	CS 17.5 mg, MMF 2000 mg, RTX (375 mg/m^2^ BSA), IVIG	IIa
13	f	31	SN	Yes	Normal	25	CS 20 mg, AZA 100 mg, RTX (375 mg/m^2^ BSA); IA/PLEX	IIIa
14	f	20	AChR	Yes	Hyperplasia	40	CS 12.5 mg, AZA 100 mg	IIIb

Characteristics of treatment-refractory patients. Treatment is shown for the timepoint patients met the definition of refractory MG

*AChR* denotes acetylcholine receptor, *AZA* azathioprine, *BSA* body surface area, *CP* Cyclophosphamide, *CS* corticosteroids, *FU* follow-up, *IA/PLEX* immunoadsorption or plasma exchange therapy (maintenance treatment), *IS* immunosuppressive, *IVIG* intravenous immunoglobulins (maintenance treatment), *MG* myasthenia gravis, *MGFA* Myasthenia Gravis Foundation of America, *MM* minimal manifestation, *MMF* mycophenolate mofetil, *MuSK* muscle-specific receptor tyrosine kinase, *NA* not applicable, *SN* seronegative, *TC* tacrolimus

Summarizing our data, we found that despite a growing number of available treatments for myasthenia gravis and improved general care, about one tenth of patients still become treatment-refractory during their course of disease with considerable implications both for patients as well as health care providers. Future studies are necessary to find potential early biomarkers for this patient group given that currently no clinical feature has a high sensitivity or specificity in predicting treatment response.

## Abbreviations

AChR: Acetylcholine receptor
CI: Confidence interval
EOMG: Early onset myasthenia gravis
IA: Immunoadsorption
IQR: Interquartile range
LOMG: Late onset myasthenia gravis
MG: Myasthenia gravis
MGFA: Myasthenia Gravis Foundation of America
MuSK: Muscle-specific tyrosine kinase
OR: Odds ratio
PLEX: Plasma exchange therapy
